# Supplementary material for: Obese mother offspring have hepatic lipidic modulation that contributes to sex-dependent metabolic adaptation later in life
Source: Commun Biol. 2021 Jan 4;4:14. doi: 10.1038/s42003-020-01513-z (PMC7782679; doi:10.1038/s42003-020-01513-z)
Supplement: Supplementary file 2 — Supplementary Information [file 42003_2020_1513_MOESM2_ESM.pdf]

Obese mother offspring have hepatic lipidic modulation that contributes to sex-dependent metabolic adaptation later in life.

Christina Savva, Luisa A. Helguero, Marcela González-Granillo, Daniela Couto, Tânia Melo, Xidan Li, Bo Angelin, Maria Rosário Domingues, Claudia Kutter and Marion Korach-André

Supplementary Table S1. List of the primer sequences used for qRT-PCR

Supplementary TableS2: Most probable PL molecular species identified from high resolution LC-MS and MS/MS acquired in positive ion mode. n=4

Supplementary TableS3: Most probable PL molecular species identified from high resolution LC-MS and MS/MS acquired in negative ion mode. n=4

Supplementary Table S1. List of the primer sequences used for qRT-PCR

Genes	**Forward primer**	**Reverse primer**
Abca1	GGAGCCTTTGTGGAACTCTTCC	CGCTCTCTTCAGCCACTTTGAG
Mdr3	TCCTCACCAAGCGACTCCGATA	ACTTGAGCAGCATCGTTGGCGA
Abcd1	TCCTGTCTGGAGGTGAGAAGCA	GCCTTCCACATCAATGCTCACG
Abcg5	CAGAGGAGATGCTTGGCTTC	TGAAAGGAACCGTGGGTAAG
Abcg8	CCTCTCAGGTGCCTTGGTTT	ACGTCGAGTAGTGAGGCTCT
Acc1	GTTCTGTTGGACAACGCCTTCAC	GGAGTCACAGAAGCAGCCCATT
Aactinb	ATGGTGGGAATGGGTCAGAA	ATCTGGGTCATCTTTTCACG
Agpat1	ACAATGGCTCCATGCTGCCCTT	GCGAGGTGAAGCGTCTTTCCTT
Agpat3	TCTGCAAACGGAAGTGGGAGGA	TGGTCTCTGTGAAGCGTGTTCC
Apob	AGCAATGTGACGGCTTCCA	TCCAGGGTGTACTGGCAAGTT
Apoe	TCGGAAGGAGCTGACTGG	CCTGCAGGCGTATTTGCT
Arg1	AGACCACAGTCTGGCAGTTG	CCACCCAAATGACACATAGG
Atgl	GGAACCAAAGGACCTGATGACC	ACATCAGGCAGCCACTCCAACA
Bsep/Abcb11	CCTTGGTAGAGAAGAGGCGACA	ATGGCTACCCTTTGCTTCTGCC
Cd36	GCCAAGCTATTGCGACATGA	TCTCAATGTCCGAGACTTTTCAA
Cd68	TGCGGCTCCCTGTGTGT	TCTTCCTCTGTTCCTTGGGCTAT
Cd80	ACCCCCAACATAACTGAGTCT	TTCCAACCAAGAGAAGCGAGG
Cds1	TTGGAGGCTTCTTTGCCAGTGG	CAAACCTGTCCATTATCCCGCC
Cerk	GGCACAAACGACGCAGAGACAT	GAAACCGTAGCCCAGCAGAGAA
Chkb/Ck	CTCTTCCGATGCTCACTACCGA	TAGAGACCGCTCTGCGAGAATG
Chrebp	GAGTGCTTGAGCCTGGCTTACA	GCTCTCCAGATGGCGTTGTTCA
Clec4f	GAGGCCGAGCTGAACAGAG	TGTGAAGCCACCACAAAAAGAG
Cpt1	AGCCTCTCCACCAGCCAGAT	CAGATGATTGGGATACTGTTTTGG
Cpt2	CAGTTCAGGAAGACAGAAGTG	CGAAGTGTCTTCAGAAACCG
Cyp7a1	TGGGCTGTGCTCTGAAGTTC	CTGTGTCCAAATGCCTTCGC
Dgat1	ACAGAGCAGATGGGGCTG	TGGGACCTGAGCCATCAT
Dgat2	CTGTGCTCTACTTCACCTGGCT	CTGGATGGGAAAGTAGTCTCGG
Dgkd	GCCTATGAGAAGACGACAGAGAG	GAGGATGAAGCCTGAGACTCGT
Elov3	ATGAATTTCTCACGCGGGTTA	GAGCTTACCCAGTACTCCTCCAA
Elovl4	ACCGTGGAGTTCTATCGCTGGA	TTTGGAACGGCTCGCGGTCTTT
Elovl5	TTCGATGCGTCACTCAGTACCT	TGTCCAGGAGGAACCATCCTT
Elovl6	GCAGAGAACACGTAGCGACT	CGCTTGTTCATCAGATGCCG
Elovl7	TCAGTCGCCAAGAGCAATGAGG	GACATGAAGGAAAGTCACTTGGC
Etnk1/Ek	TCAGTTTGCGTTGGCTTCTC	TGGTTAAAACGGACAACCGC
F4/80	CCCCAGTGTCCTTACAGAGTG	GTGCCCAGAGTGGATGTCT
Fads1	CCAGCTTTGAACCCACCAA	CATGAGGCCCATTCGCTCTA
Fas	GGAGGTGGTGATAGCCGGTAT	TGGGTAATCCATAGAGCCCAG
Gapdh	AGTCTACTGGTGTCTTCACCAC	TTGGTGGTGCAGGATGCATT
Glut2	TGGAAGGATCAAAGCAATGTTG	CACTTCGTCCAGCAATGATGA
Gpam	GCAAGCACTGTTACCAGCGATC	TGCAATCAGCCTTCGTCGGAAG
Gpd1	CGCATCACTGTGGTACAAGAGG	CTTGGTGTTGTCACCGAAGCCA
Hl	AGAGACGCAGCAAAGAATGACA	TGGAGGTCATCCAGATTTTCG
Hsl	GGAGCACTACAAACGCAACGA	TCGGCCACCGGTAAAGAG
Il-10	CGGGAAGACAATAACTGCACCC	CGGTTAGCAGTATGTTGTCCAGC
Il-18	GACTCTTGCGTCAACTTCAAGG	CAGGCTGTCTTTTGTCAACGA
Il-1b	GCAACTGTTCCTGAACTCAACT	ATCTTTTGGGGTCCGTCAACT

Il-6	TACCACTTCACAAGTCGGAGGC	CTGCAAGTGCATCATCGTTGTTC
Iplag	CTCCAGACTCTGAGGAAGTTGG	CGGCATGTGAAACAATCCCAGC
Irs1	TGTCACCCAGTGGTAGTTGCTC	CTCTCAACAGGAGGTTTGGCATG
Irs2	CCAGTAAACGGAGGTGGCTACA	CCATAGACAGCTTGGAGCCACA
Ldlr	GAATCTACTGGTCCGACCTGTC	CTGTCCAGTAGATGTTGCGGTG
Lxrα	GAGTTGTGGAAGACAGAACCTCAA	GGGCATCCTGGCTTCCTC
Lxrβ	CCCCACAAGTTCTCTGGACAC	TGGCGGAGGTACTGGGC
Me	AGTATCCATGACAAAGGGCAC	ATCCCATTACAGCCAAGGTC
Mrc1	TGATTACGAGCAGTGGAAGC	GTTCACCGTAAGCCCAATTT
Pcsk9	ATGGCACCAGACAGAGGAAGAC	CACGCTGTTGAAGTCGGTGATG
Pcyt1a/Ct	TCCTTCCAAAGTGCAGCGTTGC	GCAGGCTTCTTCCATAGTCACC
Pcyt2/Et	GGAAGACAGAGATTGTACCCGAC	GCACAATCAGGTCTGTAGTGAGG
Pemt	CCACTGCTTCACACAGGCTA	CAAGGCCCAGGAAGTAGGTG
Pepck	CCACAGCTGCTGCAGAACA	GAAGGGTCGCATGGCAAA
Pgs1	CTGGACTTCACCAGAGGCTCAA	CCGATGGTCTCATTGAAGCGCT
Pla2g12a	GCAAGAACGACTGTGACGAGGA	GATGACGCTGTCAAAGAGGAGC
Pla2g7/Lppla2	CTTCAAGCCCTTAGTGAGGACC	TGCGATGTCCTTTGGAGTCTGG
Pld1	CTGGACTGCATCCTCAAACGGA	TCATCAACGTCCGCTTGCTGTAC
Ppap2a	CAGTTGGACTCATTCAGGGAGC	TGGCGGTTTCATGGAGAGTCGT
Ppara	ACAACCCGCCTTTTGTCA	AAGGATGTGGGGAGAGCC
Ptdss2	CTACGACGATGGCACTAACACC	GTGTTGTAGGCTGTATCCTGAGG
Ptplb	GGTGAGAGCATACCTGGCTAAG	GTCAGGACAACAGAAGATGGCAC
Scd1	GCAAGCTCTACACCTGCCTCTT	CGTGCCTTGTAAGTTCTGTGGC
Scd2	GTCTGACCTGAAAGCCGAGAAG	GCAAGAAGGTGCTAACGCACAG
Sgms1	GCATAGTTGGCACGCTGTACCT	TAAGCCACCTCCAGCAATGAGC
Sgms2	GCACTTCCAGTGTGCTCCCAAG	AAGTCTCCGCACAGGATGTGAG
Smpd1/Smase	AACTCTGAGCCGACCACTAGCT	GTCCAGGACCACATGAGAGCTT
Socs3	ATGGTCACCCACAGCAAGTTT	TCCAGTAGAATCCGCTCTCCT
Srebp1	CGACTACATCCGCTTCTTGCAG	CCTCCATAGACACATCTGTGCC
Srebp2	AGAAAGAGCGGTGGAGTCCTTG	GAACTGCTGGAGAATGGTGAGG
Trl4	ATGGCATGGCTTACACCACC	GAGGCCATTTTTGTCTCCACA

Suppl. Table S2: Most probable positive PL molecular species identified by LC-MS

(orange highlight) internal standard

PL classes	ID	m/z [M+H]+	FA chains
Ceramides	Cer(d34:1)	538,5199	Cer(16:0/d18:1)
	Cer(d35:1)	552,5356	
	Cer(d36:1)	566,5512	Cer(18:0/d18:1)
	Cer(d38:1)	594,5825	Cer(20:0/d18:1)
	Cer(d40:1)	622,6138	Cer(22:0/d18:1)
	Cer(d40:2)	620,5982	Cer(22:0/d18:2)
	Cer(d42:1)	650,6451	Cer(24:0/d18:1)
	Cer(d42:2)	648,6295	Cer(24:1/d18:1)
	GlcCer(d18:1/22:0)/GlcCer(d16:1/24:0)/GalCer(d18:1/22:0)	784,6666	
LysoPC	LPC(16:0)	496,3403	
	LPC(16:1)	494,3247	
	LPC(18:0)	524,3716	
	LPC(18:1)	522,3560	
	LPC(18:2)	520,3403	
	LPC(18:3)	518,3247	
	LPC(19:0)	538,3873	
	LPC(20:1)	550,3873	
	LPC(20:3)	546,3560	
	LPC(20:4)	544,3403	
	LPC(20:5)	542,3247	
	LPC(22:6)	568,3403	
LysoPE	LPE(16:0)	454,2934	
	LPE(16:1)	452,2777	
	LPE(18:0)	482,3247	
	LPE(18:1)	480,3090	
	LPE(18:2)	478,2934	
	LPE(20:1)	508,3403	
	LPE(20:4)	502,2934	
	LPE(20:5)	500,2777	
	LPE(22:4)	530,3247	
	LPE(22:6)	526,2934	
	LPE(P-16:0)	438,2985	
	LPE(P-18:0)/LPE(O-18:1)	466,3298	
Phosphatidylcholine (PC)	PC(28:0)	678,5074	14:0/14:0
	PC(30:0)	706,5387	
	PC(32:0)	734,5700	16:0/16:0
	PC(32:1)	732,5543	16:0/16:1
	PC(32:2)	730,5387	16:016:2
	PC(34:1)	760,5856	16:0/18:1
	PC(34:2)	758,5700	16:0/18:2
	PC(34:3)	756,5543	16:1/18:2
	PC(34:4)	754,5387	
	PC(36:1)	788,6169	18:0/18:1

	PC(36:2)	786,6013	18:0/18:2; 18:1/18:1
	PC(36:3)	784,5856	16:0/20:3; 18:1/18:2
	PC(36:4)	782,5700	16:0/20:4
	PC(36:5)	780,5543	16:1/20:4
	PC(36:6)	778,5387	
	PC(38:4)	810,6013	18:0/20:4
	PC(38:5)	808,5856	
	PC(38:6)	806,5700	16:0/22:6
	PC(38:7)	804,5543	16:1/22:6
	PC(38:8)	802,5387	
	PC(40:4)	838,6326	20:0/20:4
	PC(40:5)	836,6169	
	PC(40:6)	834,6013	18:0/22:6
	PC(40:7)	832,5856	18:1/22:6
	PC(40:8)	830,5699	20:4/20:4; 18:2/22:6
	PC(40:9)	828,5543	
	PC(42:10)	854,5700	
	PC(42:11)	852,5543	
	PC(42:6)	862,6326	
	PC(42:7)	860,6169	
	PC(42:9)	856,5856	
**Alkylacyl-PC**	PC(O-32:0)	720,5907	
	PC(O-34:1)/PC(P-34:0)	746,6064	16:0; 18:1
	PC(O-36:3)/PC(P-36:2)	770,6064	
	PC(O-36:4)/PC(P-36:3)	768,5907	20:4
	PC(O-36:5)/PC(P-36:4)	766,5751	
	PC(O-38:4)/ PC(P-38:3)	796,6220	20:4
	PC(O-38:5)/PC(P-38:4)	794,6064	
	PC(O-38:6)/PC(P-38:5)	792,5907	22:6
**Phosphatidylethanolamine (PE)**	PE(28:0)	636,4604	14:0/14:0
	PE(30:3)	658,4448	
	PE(32:0)	692,5230	
	PE(32:1)	690,5074	16:0/16:1
	PE(34:1)	718,5387	16:0/18:1
	PE(34:2)	716,5230	16:0/18:2
	PE(34:3)	714,5074	16:1/18:2
	PE(34:4)	712,4917	16:1/18:3; 18:2/18:2
	PE(36:1)	746,5700	18:0/18:1
	PE(36:2)	744.554 3	18:0/18:2; 18:1/18:1
	PE(36:4)	740,5230	18:2/18:2
	PE(38:2)	772,5856	18:1/20:1
	PE(38:4)	768,5543	18:0/20:4

	PE(38:5)	766,5387	
	PE(38:6)	764,5230	16:0/22:6
	PE(38:8)	760,4917	
	PE(40:10)	784,4917	
	PE(40:4)	796,5856	18:0/20:4
	PE(40:6)	792,5543	18:0/22:6
	PE(40:7)	790,5387	18:1/22:6
	PE(40:8)	788,5230	20:4/20:4
	PE(40:9)	786,5074	
	PE(42:10)	812,5230	
	PE(42:6)	820,5856	22:2/20:4
	PE(42:7)	818,5700	20:1/22:6
	PE(42:9)	814,5387	
**Alkylacyl-PE**	PE(P-42:6)/PE(O-42:7)	804,5907	20:4
	PE(P-42:2)/PE(O-42:3)	812,6556	
	PE(P-36:1)/PE(O-36:2)	730,5751	18:2; 18:1
	PE(P-36:2)/PE(O-36:3)	728,5594	18:2; 18:1
	PE(P-36:4)/PE(O-36:5)	724,5281	20:4
	PE(P-38:3)/PE(O-38:4)	754,5751	20:4
	PE(P-38:4)/PE(O-38:5)	752,5594	20:4; 22:4
	PE(P-38:5)/PE(O-38:6)	750,5438	20:4; 22:5
	PE(P-38:7)/PE(O-38:8)	746,5110	
	PE(P-40:3)/PE(O-40:4)	782,6064	20:4; 22:3
	PE(P-40:4)/PE(O-40:5)	780,5907	20:4
	PE(P-40:5)/PE(O-40:6)	778,5751	22:6; 22:4
	PE(P-40:6)/PE(O-40:7)	776,5594	18:0; 20:4; 22:6
	PE(P-40:8)/PE(O-40:7)	774,5438	22:6
**Sphingomyelin (SM)**	SM(d34:1)	703,5754	
	SM(d35:1)	717,5911	
	SM(d36:1)	731,6067	
	SM(d38:1)	759,6380	
	SM(d40:1)	787,6693	
	SM(d40:2)	785,6537	
	SM(d42:1)	815,7006	
	SM(d42:2)	813,6850	
	SM(d42:3)	811,6693	

Suppl.Table S3: Most probable negative PL molecular species identified by LC-MS

(highlighted) internal standard

PL classes	ID	m/z [M-H]-	FA chains
Cardiolipin (CL)	CL(56:0)	1239,8390	
	CL(70:5)	1425,9800	
	CL(70:6)	1423,9640	
	CL(70:7)	1421,9480	
	CL(72:6)	1451,9960	
	CL(72:7)	1449,9800	
	CL(72:8)	1447,9640	
	CL(74:10)	1471,9640	
	CL(74:8)	1475,9960	
	CL(74:9)	1473,9800	
	CL(76:11)	1497,9800	
	CL(76:12)	1495,9640	
LysoPI	LPI(16:0)	571,2883	
	LPI(18:0)	599,3196	18:0
	LPI(20:4)	619,2883	
Phosphatidylglycerides (PG)	PG(28:0)	665,4394	14:0/14:0
	PG(32:1)	719,4863	16:0/16:1
	PG(34:1)	747,5176	16:0/18:1
	PG(34:2)	745,5020	16:1/18:1
	PG(36:2)	773,5333	18:1/18:1
	PG(36:3)	771,5176	18:1/18:2
	PG(36:4)	769,5020	16:0/20:4; 18:2/18:2
	PG(38:4)	797,5333	
	PG(38:5)	795,5176	18:1/20:4
	PG(38:6)	793,5020	18:2/20:4
	PG(38:7)	791,4863	16:1/22:6
	PG(40:5)	823,5489	18:1/22:4
	PG(40:6)	821,5333	
	PG(40:7)	819,5176	18:1/22:6
	PG(40:8)	817,5020	20:4/20:4; 18:2/22:6
	PG(42:10)	841,5020	20:4/22:6
	PG(42:8)	845,5333	
	PG(42:9)	843,5176	
	PG(44:12)	865,5020	22:6/22:6
Phosphatidylinositol (PI)	PI(32:0)	809,5180	16:0/16:0
	PI(34:1)	835,5337	
	PI(34:2)	833,5180	16:0/18:2
	PI(36:1)	863,5650	
	PI(36:2)	861,5493	18:0/18:2
	PI(36:3)	859,5337	

	PI(36:4)	857,5180	16:0/20:4
	PI(38:3)	887,5650	
	PI(38:4)	885,5493	18:0/20:4
	PI(38:5)	883,5337	18:1/20:4
	PI(38:6)	881,5180	16:0/22:6
	PI(40:4)	913,5806	18:0/22:4
	PI(40:5)	911,5650	
	PI(40:6)	909,5493	18:0/22:6

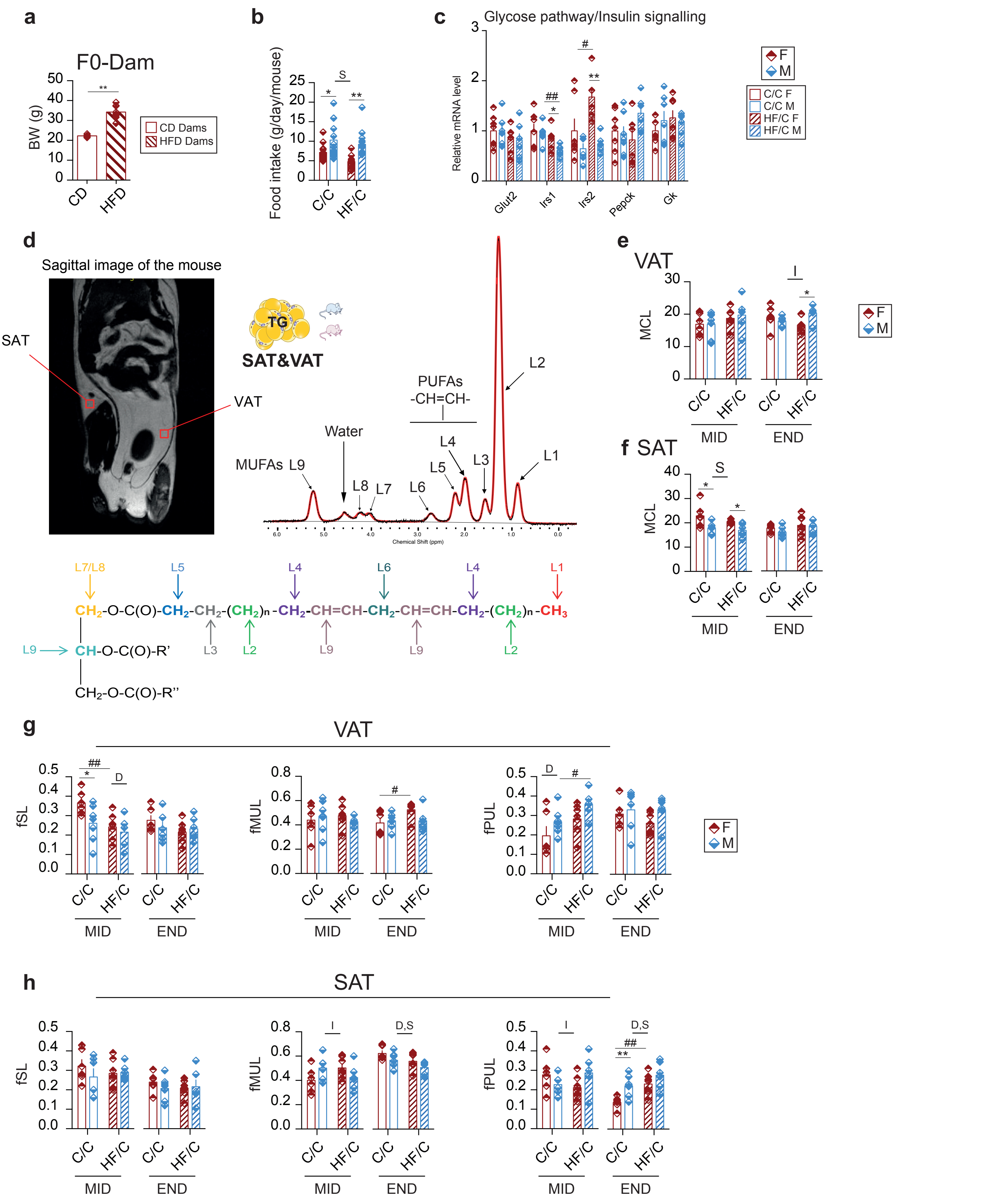

Supplementary Figure S1. **a** Body weight of F0 dam fed the CD (open bars) and the HFD (stripped bars); **b** Food intake and **c** Relative expression level of genes from the glucose and insulin signaling pathways in F (red bars) and M (blue bars) offspring born from CD mother (open bars) and HFD mother (stripped bars); **d** Sagittal image of the whole body fat mouse and one representative MRS spectrum for *in vivo* quantification of TG composition in adipose tissue; Mean chain lenght (MCL) in **e** VAT and **f** SAT; Fraction of saturated lipids (fSL), monounsaturated lipids (fMUL) and polyunsaturated lipids (fPUL) in **g** VAT and **h** SAT in F (red bars) and M (blue bars) offspring born from CD mother (C/C, open bars) and HFD mother (HF/C, stripped bars). For **a** CD F0-dams n=6 and HFD F0-dams n=6. For **b** C/C F n=11 and C/C M n=12, for HF/C F n=11 and HF/C M n=10. For **c** C/C F n=6 and C/C M n=7, for HF/C F n=7 and HF/C M n=6. For **d** n=1. For **e-h** C/C F n=6 and C/C M n=7, for HF/C F n=7 and HF/C M n=6. Data are presented as mean ± sem. Two-way ANOVA (sex (S), mother diet (D), interaction (I) between sex and diet, and (ns) for not significant) followed by a Tukey's multiple comparisons test when significant ($p<0.05$). *, M *vs* F and #, HF/C *vs* C/C ($p<0.05$), ** or ##, $p<0.01$.

TG species detected by LC-MS in offspring's liver.

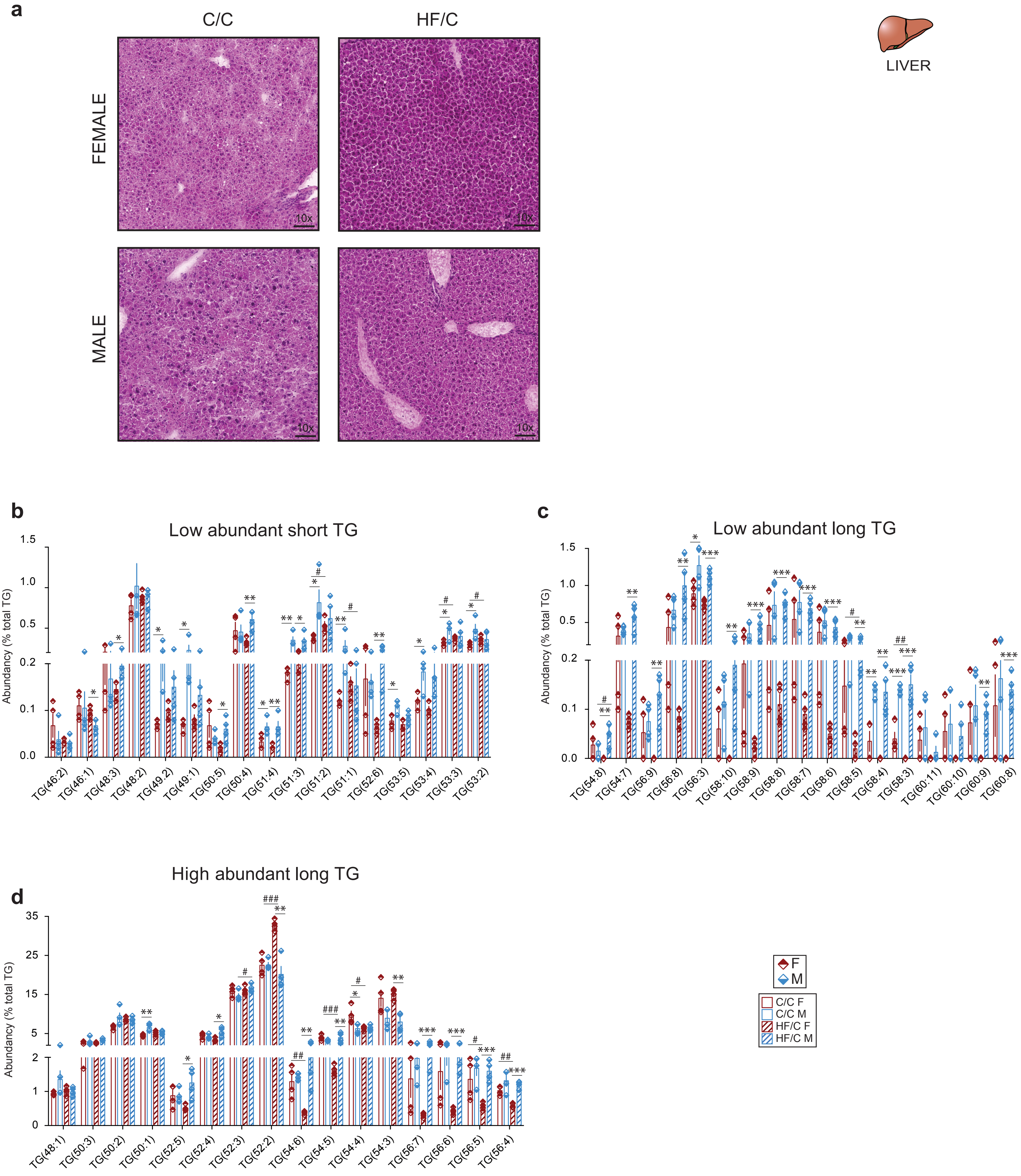

Supplementary Figure S2. **a** Representative H&E staining of the liver; Hepatic relative abundance of TG species classified as **b** low abundant short chain TG; **c** low abundant long chain TG and **d** high abundant long chain TG in C/C (open bars) and HF/C (stripped bars) in F (red bars) and M (blue bars) offspring. For **a-d** n=4 per group. Data are presented as mean ± sem. Unpaired two-tailed Student's *t*-test corrected for multiple comparisons using the Holm-Sidak method, with alpha=5.000%. *, M *vs* F and #, HF/C *vs* C/C (p<0.05), ** or ##, p<0.01 and *** or ###, p<0.001.

Relative expression of genes in offspring's liver.

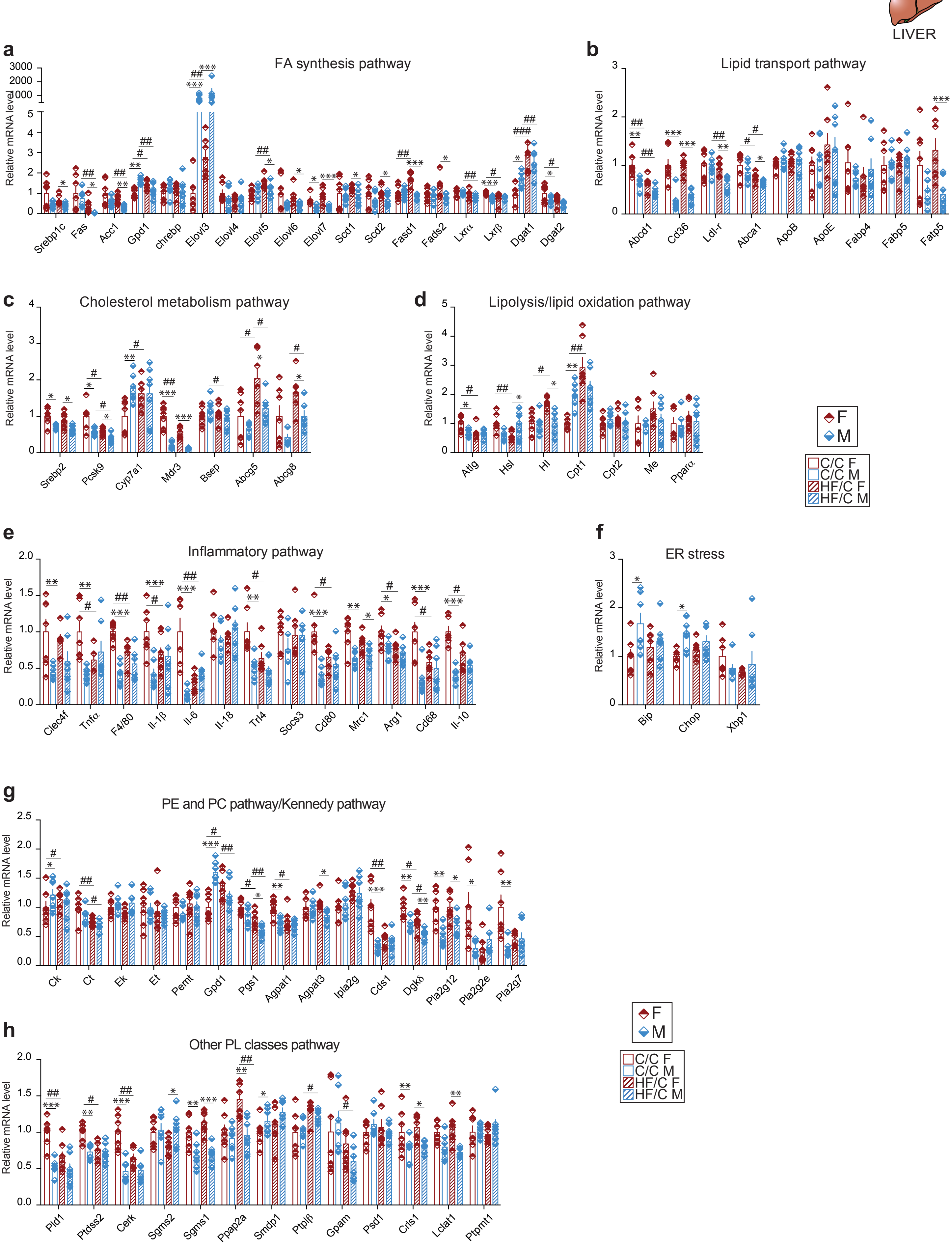

Supplementary Figure S3. Relative hepatic levels of genes involved in the **a** Fatty acid synthesis pathway; **b** Lipid transport pathway; **c** Cholesterol metabolism pathway; **d** Lipolysis and lipid oxidation pathways; **e** Inflammatory pathway; **f** Endoplasmic reticulum (ER) stress pathway; **g** PE and PC / Kennedy pathway and **h** Other PL class pathway in C/C (open bars) and HF/C (stripped bars) in F (red bars) and M (blue bars) offspring. For **a-h** n=7 per group. Data are presented as mean ± sem. Unpaired two-tailed Student's *t*-test corrected for multiple comparisons using the Holm-Sidak method, with alpha=5.000%. *, M *vs* F and #, HF/C *vs* C/C ($p<0.05$), ** or ##, $p<0.01$ and *** or ###, $p<0.001$.

Relative abundance of the PC and PE species detected by LC-MS in offspring's liver.

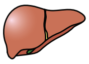

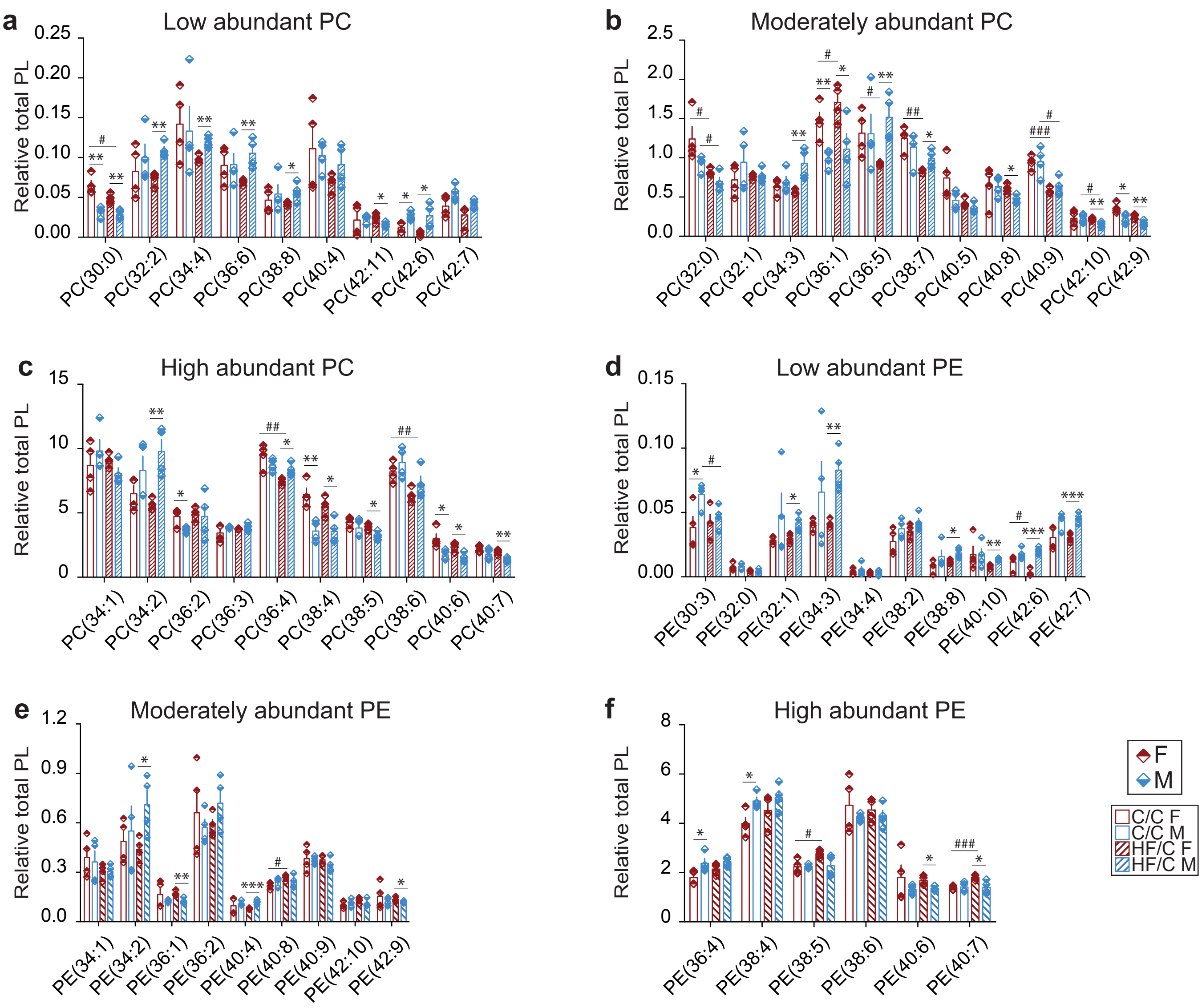

Supplementary Figure S4. Relative hepatic content of **a** Low, **b** Moderate and **c** High abundant phosphatidylcholine (PC) species and ; **d** Low, **e** Moderate and **f** High abundant phosphatidylethanolamine (PE) species in C/C (open bars) and HF/C (stripped bars) in F (red bars) and M (blue bars) offspring. For **a-f** n=4 per group.
Data are presented as mean ± sem. Unpaired two-tailed Student's *t*-test corrected for multiple comparisons using the Holm-Sidak method, with alpha=5.000%. *, M *vs* F and #, HF/C *vs* C/C ($p<0.05$), ** or ##, $p<0.01$ and *** or ###, $p<0.001$.

Relative abundance of the LysoPC, LysoPE, PG and CL species detected by LC-MS in offspring's liver

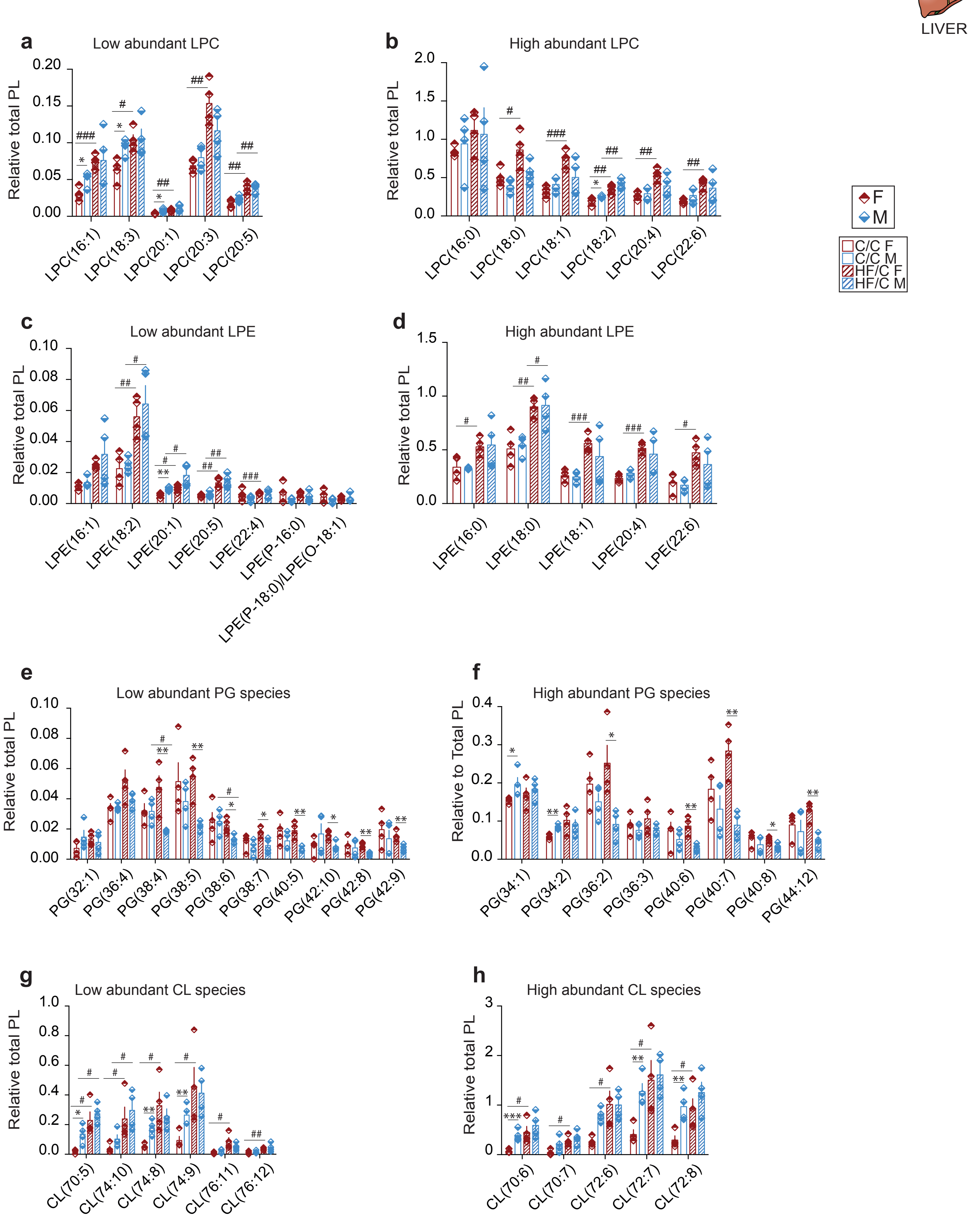

Supplementary Figure S5. Relative hepatic content of **a** Low, **b** High abundant lysophosphatidylcholine (LPC) species ; **c** Low and **d** High abundant lysophosphatidylethanolamine (LPE) species; **e** Low and **f** High abundant phosphatidylglycerides (PG) and **g** Low and **h** High abundant cardiolipin (CL) species in C/C (open bars) and HF/C (stripped bars) in F (red bars) and M (blue bars) offspring. For **a-h** n=4 per group. Data are presented as mean ± sem. Unpaired two-tailed Student's *t*-test corrected for multiple comparisons using the Holm-Sidak method, with alpha=5.000%.
*, M *vs* F and #, HF/C *vs* C/C (p<0.05), ** or ##, p<0.01 and *** or ###, p<0.001.

Relative abundance of the Cer, SM and PI species detected by LC-MS in offspring's liver

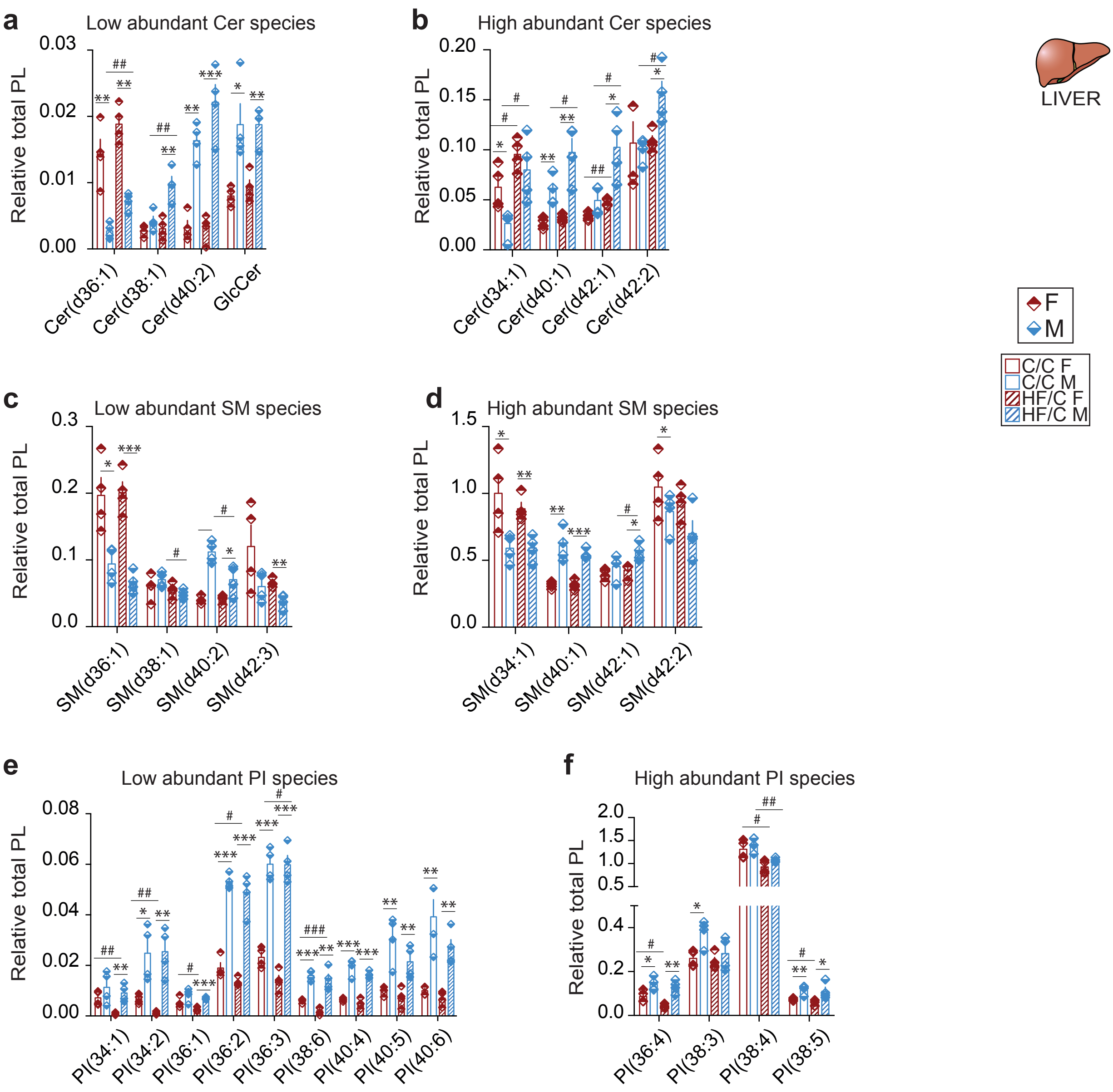

Supplementary Figure S6. Relative hepatic content of **a** Low and **b** High abundant Ceramides (Cer) species ; **c** Low and **d** High abundant sphingomyelin (SM) species and **e** Low and **f** High abundant phosphatidylinositol (PI) species in C/C (open bars) and HF/C (stripped bars) in F (red bars) and M (blue bars) offspring.
For **a-f** n=4 per group. Data are presented as mean ± sem. Unpaired two-tailed Student's *t*-test corrected for multiple comparisons using the Holm-Sidak method, with alpha=5.000%.
*, M *vs* F and #, HF/C *vs* C/C (p<0.05), ** or ##, p<0.01 and *** or ###, p<0.001.
